# Superheated Compressed Air and Comminuted Medicated Vapours in the Treatment of Diseases of the Respiratory Tract

**Published:** 1904-11-19

**Authors:** Ivan Tolniewski


### SUPERHEATED COMPRESSED AIR AND COMMINUTED MEDICATED VAPOURS IN THE TREATMENT OF DISEASES OF THE RESPIRATORY TRACT.

By Ivan Tolniewski, M.D.

The author, who has been for some years engaged in studying the effect of open-air and other methods of treatment of pulmonary tuberculosis, has sent us a long article on the above subject. The following are some of the chief points of interest.

The only rational method of administering medicaments intended to act locally on the respiratory tract is by inhalation, as their direct and primary action is thus secured, uninfluenced by chemical changes, and without disturbance of digestion or secretion. The author admits that inhalations have proved of little efficacy in the past, but claims that this has been due to the faulty method of administration employed. He has devised an apparatus which he believes overcomes many of the objections to treatment by inhalation. The fluid employed may be either volatile or non-volatile, as the comminution is so perfect that either may be inhaled to the utmost limits of tidal respiration, and pass by diffusion to the air-sacs. The apparatus is constructed entirely of glass and aluminium, and is therefore easily sterilisable. The comminuting tube has the usual hole at the side, but is so arranged that when the air is turned off the hole becomes submerged before the instrument is laid aside and clogging of the comminutor becomes impossible. Also by turning on only a little compressed air, the hole remains partially submerged, and part of the air keeps the solution agitated, thus avoiding precipitation. The apparatus is also fitted with an adjustable vibratory valve, whereby a vibratory impulse can be given to the vapour, which may be conveyed to the lungs or nasal cavities and sinuses. Dr. Tolniewski claims that this acts as a vibratory massage, and that he has found it of advantage in the treatment of diseases of the respiratory tract and of the ears. The vibrations may be made slowly or quickly, at the will of the operator. There is a mixing-chamber, consisting of a horizontal glass cone, so arranged that the tubes of two or more comminuting flasks impinge upon it, so that the physician may alter the nature of the medicaments at will. Hitherto the mixing arrangement in comminutors has been very imperfect, and the medicament in one flask was apt to be contaminated by condensed vapour from another, but the tubes in Dr. Tolniewski's apparatus being placed at an angle of 84° to the flasks, all condensed vapour returns automatically to its own bottle. Back pressure, a common defect in older devices, is avoided by an outward valve on the end of each conducting-tube. Contamination of the solutions when more than one is used is thus avoided. The apparatus is also provided with an electric heater, worked by the ordinary lighting current. By this means a warm vapour instead of a cold one can be used, which gives a great advantage as regards clinical results. The heater consists of an air-tight tube containing a heating coil. A nickel-plated hood packed with asbestos surrounds it. The compressed air enters at the bottom of the hood and issues at the top, and in its passage any temperature up to 400° F. can be communicated to it. A special delivery tube for superheated air is attached to the generator. There is also an accessory apparatus for the production of practically unlimited quantities of ozone.

The application of vibrating warm vapour under pressure adds very materially to the action of the drugs in reducing congestion, restoring normal circulation, and in favouring absorption of exudates. It tends to free the canals leading to the various sinuses when obstructed by congestion or thickening of the mucosa, and carries the medicament to places that are often the seat of catarrhal conditions.

Dr. Tolniewski has found this method of much help in the treatment of diseases of the middle ear as it assists in overcoming adhesions, rigidity of the ossicles and contraction of ligaments much more perfectly and with infinitely less risk to the patient than the other forms of vibratory massage at present in use.

It is, however, in pulmonary affections that this treatment finds its greatest field of usefulness and especially in pulmonary tuberculosis. While it may not be possible in this way to destroy the bacilli a strong inhibitory action is produced and the surrounding tissues are fortified against the advance of the disease into new areas. It is therefore a useful adjuvant to the open-air treatment and other measures calculated to combat the disease by improving the general nutrition. The author adds that his investigations with regard to the effect of this treatment in pulmonary tuberculosis are still incomplete, but he is carrying out a series of observations on a number of cases and his results when complete will form the subject of a further communication.

